# COVID-ConvNet: A Convolutional Neural Network Classifier for Diagnosing COVID-19 Infection

**DOI:** 10.3390/diagnostics13101675

**Published:** 2023-05-09

**Authors:** Ibtihal A. L. Alablani, Mohammed J. F. Alenazi

**Affiliations:** Department of Computer Engineering, College of Computer and Information Sciences, King Saud University, Riyadh P.O. Box 11451, Saudi Arabia

**Keywords:** COVID-19, chest X-ray, coronavirus, deep learning, CNN

## Abstract

The novel coronavirus (COVID-19) pandemic still has a significant impact on the worldwide population’s health and well-being. Effective patient screening, including radiological examination employing chest radiography as one of the main screening modalities, is an important step in the battle against the disease. Indeed, the earliest studies on COVID-19 found that patients infected with COVID-19 present with characteristic anomalies in chest radiography. In this paper, we introduce COVID-ConvNet, a deep convolutional neural network (DCNN) design suitable for detecting COVID-19 symptoms from chest X-ray (CXR) scans. The proposed deep learning (DL) model was trained and evaluated using 21,165 CXR images from the COVID-19 Database, a publicly available dataset. The experimental results demonstrate that our COVID-ConvNet model has a high prediction accuracy at 97.43% and outperforms recent related works by up to 5.9% in terms of prediction accuracy.

## 1. Introduction

**Figure 1:**
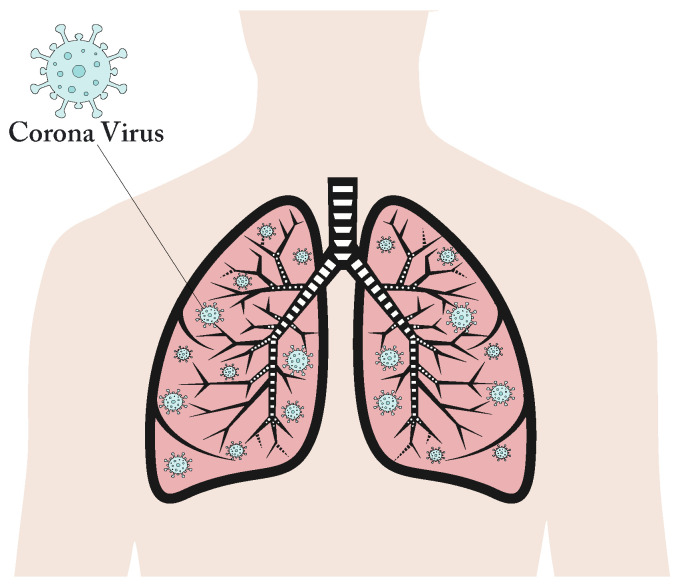
Illustration of how COVID-19 affects human lungs.

The most recent viral pandemic, COVID-19, arose in the Chinese city of Wuhan [1,2]. Because the outbreak surged across the globe and infected millions of individuals, the WHO declared it a worldwide pandemic, and the number of affected people continues to rise daily. As of 22 September 2022, over 610 million coronavirus cases worldwide, as well as 6.5 million deaths, had been reported [3]. The COVID-19 virus primarily spreads through respiratory droplets when an infected person coughs, sneezes, talks, or breathes [4]. Symptoms of COVID-19 can range from mild to severe and can include fever, cough, shortness of breath, and loss of taste or smell [5]. The COVID-19 pandemic has had a significant impact on public health, economies, and social systems around the world. Many countries have implemented measures such as lockdowns, travel restrictions, and vaccination campaigns to control the spread of the virus [6]. The pandemic has also highlighted the importance of public health infrastructure, scientific research, and global cooperation in responding to infectious diseases. Figure 1 shows how the COVID-19 virus spreads to human lungs and causes pneumonia, resulting in serious harm.

Currently, no treatment directly interacts with this new type of coronavirus. Many medley medicines, comprised mainly of varying concentrations of ethanol, hydrogen peroxide, and isopropyl alcohol, have been developed by certain firms in response to the unique virus. The WHO has confirmed and approved the use of these treatments worldwide [7].

The evolution of computer vision diagnostic tools for the treatment of COVID-19 would give medical professionals an automated “second reading”, helping in the critical diagnosis of COVID-19 infected patients and improving the decision-making process to cope with this widespread illness. Radiological examination, including chest X-rays and computed tomography (CT) scans, has played an important role in the screening and diagnosis of COVID-19 [8]. Chest X-rays are often used as a first-line imaging tool in the initial screening of suspected COVID-19 patients, while CT scans are typically reserved for more severe cases or for patients with inconclusive chest X-ray results [9]. Radiological examination can also help monitor the progression of the disease and assess the effectiveness of treatment [10]. Radiologists and other medical professionals may indeed find it challenging to differentiate between pneumonia caused by COVID-19 and other kinds of viral and bacterial pneumonia based only on diagnostic imaging. X-ray imaging is an easy and affordable technique for identifying lung and COVID-19 infections. In X-ray scans, opacities or patchy infiltrates, similar to other viral pneumonia symptoms, are frequently detected in COVID-19-infected patients. However, on X-ray images, earlier stages of COVID-19 do not seem to show any abnormalities. COVID-19 affects the mid and upper or lower areas of the lungs and develops patchy infiltrations, typically with evidence of consolidation, as the patient’s condition worsens.

All facets of modern life, including business, marketing, the military, communications, engineering, and health, rely on innovative technology applications. The medical industry requires the extensive use of new technologies, from precisely describing symptoms to accurately diagnosing conditions and conducting examinations of patients [2]. The ability of artificial intelligence (AI) and DL algorithms to accurately recognize COVID-19 might be viewed as a supporting factor to improve conventional diagnostic approaches, such as chest X-rays [11]. DL and CNN models have excelled in a large number of medical image categorization applications [12].

Deep learning research on the use of chest X-rays to detect COVID-19 symptoms has shown promising results. Several studies have used deep learning algorithms to develop models that can accurately identify COVID-19 cases based on chest X-ray images. These models typically use convolutional neural networks (CNNs) to extract features from chest X-ray images and classify them as positive or negative for COVID-19. Some studies have also used transfer learning, a technique that uses pre-trained CNNs to improve the accuracy and efficiency of the model.

One potential limitation of these models is the lack of large, diverse datasets for training and validation. This may limit the generalizability of the trained models and can lead to overfitting, where the model performs well on the training data but poorly on new, unseen data. In addition, some studies have reported high false-positive rates or difficulties in distinguishing COVID-19 from other respiratory illnesses. Moreover, some related works demand a large number of training parameters and complex computational resources, making them challenging to implement in real-world scenarios, especially in the healthcare industry. In our study, we overcome the limitations of previous studies by using a large database that includes a significant number of chest X-ray scans (21,165 images) to improve the accuracy and generalizability of the trained model by providing more diverse samples; this can help to reduce overfitting problems. Furthermore, complexity is avoided by reducing the number of parameters and computational resources required by the proposed CNN model without sacrificing accuracy.

In this article, our major contributions are the following:

1. We propose a deep learning approach, COVID-ConvNet, to help in the early diagnosis of COVID-19 cases.
2. We employ conventional chest X-rays for the identification and diagnosis of COVID-19 while empirically evaluating the proposed deep learning image classifiers. Three experimental classifications were performed with four, three, and two classes.
3. We compare the results of various DL models to show the COVID-19 classification results and to demonstrate the superiority of the proposed model.

The rest of the paper is organized as follows. Section 2 reviews related works on the detection of the COVID-19 virus based on machine learning (ML) methods. Section 3 describes our proposed method, COVID-ConvNet. Section 4 presents the experimental results obtained using this method. Finally, Section 5 presents a conclusion of the article.

## 2. Related Works

This section relates recent works on the diagnosis of the COVID-19 virus, in which applied ML and DL techniques are discussed.

In [13], Ohata et al. proposed a transfer learning model to train several previously pre-trained DL models to precisely predict COVID-19 cases. Two datasets were used, containing 194 X-ray images of both coronavirus-infected and healthy patients. Due to the lack of publicly available images of COVID-19 patients, the transfer learning concept was used for this task. CNNs were trained using a variety of architectures and integrated with conventional machine learning approaches, including k-nearest neighbors, support-vector machines (SVM), random forest, Bayes, and multilayer perceptrons (MLP). The results showed that the MobileNet architecture with the SVM classifier employing a linear kernel was the best classifier pair for one of the datasets.

Tabik et al. introduced a framework known as the COVID smart-data-based network (COVID-SDNet) in [14], in addition to a dataset called COVIDGR-1.0. The COVIDGR-1.0 dataset has two categories: positive and negative. It contains 852 images, 426 of which are positive and 426 of which are negative. The results showed that the COVID-SDNet model achieved good and consistent results in the case of medium and high severity levels. Yet, it showed low accuracy in mild and normal polymerase chain reaction (PCR)+ severity levels. However, Ohata et al.’s method and COVID-SDNet are rather time-consuming [15].

Wang et al. put forward the COVID-Net model in [16], a CNN method designed to detect COVID-19 infection based on X-ray images of human chests. A dataset from five open-access data repositories called COVIDx was introduced, containing 13,975 CXR scans from 13,870 cases. Based on the results, the COVIDx dataset has the most COVID-19-positive patient cases of any open-access benchmark dataset. This dataset presents three classes, i.e., normal, phenomena, and COVID-19. According to the evaluation results, the confusion matrix reveals that the accuracy of the COVID-Net model for the detection of the COVID-19 virus reaches 96%.

In [17], Hemdan et al. developed a new model called COVIDX-Net. The public dataset of X-ray photos used in this study for identifying negative and positive COVID-19 cases was reported by Rosebrock and Cohen and is available on the GitHub repository [18]. Their dataset consists of 50 X-ray images, 25 of which are normal and 25 of which are COVID-19-positive cases. VGG19, residual network (ResNetV2), InceptionV3, DenseNet121, Inception ResNetV2, Xception, and MobileNetV2 form the foundation of the COVIDX-Net framework. The results obtained confirmed that the DenseNet201 and VGG19 models achieved the best performance scores of the deep learning classifiers, with an accuracy of 90%. The COVID-Net and COVIDX-Net methods, on the other hand, suffered from overfitting and were difficult to use in practical systems [15].

Arias-Londoño et al. proposed in [19] a method for the automatic detection of COVID-19 (AD-COVID19) employing a deep neural network (DNN) based on chest X-ray images with a segmentation method. Their method for training the CNN was to use a total of about 79,500 X-ray images, including more than 8500 COVID-19 case reports collected from various sources. The automatic COVID-19 diagnosis tool distinguished between groups of controls, pneumonia, and COVID-19. Three experiments were performed using various preprocessing schemes to compare and evaluate the models developed by the authors. The objective was to determine the way data preprocessing impacted the data and enhanced its explanatory capacity on results. An important investigation of diverse variability problems affecting the system and its impacts was also applied. The proposed methodology achieved a prediction accuracy of 91.5%, with an average recall of 87.4% for the worst and most coherent investigation that needed a prior automated segmentation of the lung area.

In [20], Wang et al. used a previous residual learning method to accurately classify COVID-19 infections. It is a basic model to diagnose COVID-19, built thanks to 3D chest CT scans. Their algorithm can indeed predict whether a CT image exhibits pneumonia and can distinguish between COVID-19-caused pneumonia and interstitial lung disease (ILD) caused by other viruses. A couple of ResNet models based on branches were embedded into a model architecture for end-to-end training by constructing a prior-attention residual learning (PARL) block. The model was assessed using confusion matrices on an offline-testing dataset of 600 photos (i.e., 200 images per class). The results depict that the suggested approach can diagnose COVID-19 effectively. However, the proposed model has many parameters, making it difficult to implement in practical applications, particularly in the healthcare field [15].

In [21], Nikolaou et al. introduced a CNN model with a dense layer on top of a pre-trained baseline convolutional network. According to the experimental data, for a two-class classification problem that included COVID-19 and healthy lungs, this model had a 95% accuracy rate, whereas for a three-class classification problem, which including COVID-19-infected lungs, normal lungs, and other viral pneumonia, accuracy reached 93%.

Ismael et al. [22] proposed DL-based methods that aimed to classify COVID-19, as well as normal chest X-ray scans; these methods included the fine-tuning of a pre-trained CNN, deep feature extraction, and end-to-end training of a constructed CNN model. This study employed 200 normal and 180 COVID-19 patients’ chest X-ray scans. The experimental results indicated that, with an accuracy score of 94.7%, the ResNet50 model surpassed the other pre-trained CNN models.

In [23], Narin et al. developed five pre-trained CNN-based models (i.e., ResNet101, ResNet50, InceptionV3, ResNet152, and Inception-ResNetV2) to find symptoms of coronavirus pneumonia on chest X-ray scans. Categories included COVID-19, as well as normal, viral, and bacterial pneumonia. Three datasets were used to perform the experiments. The open-source GitHub repository contained 341 COVID-19 patients’ chest X-ray scans [18]. Additionally, The Kaggle dataset “Chest X-ray data (pneumonia)” includes 2772 bacterial and 1493 viral pneumonia chest X-rays [24]. Furthermore, 2800 normal chest X-ray scans from the “ChestX-ray8” dataset [25] were selected. Amongst the five tested models, ResNet50 and ResNet101 showed the best accuracy, i.e., 96.1%.

In [26], Abbas et al. proposed a deep CNN model named “decompose, transfer, and compose” to classify COVID-19 chest X-rays (DeTraC). Normal CXR images were collected from the Japanese Society of Radiological Technology (JSRT) image data collection [27]. COVID-19 images, as well as severe acute respiratory syndrome (SARS) images, were collected from the COVID-19 data collection [28]. Experimental results revealed that the DeTraC model detected COVID-19 X-ray scans from healthy and SARS cases with a high accuracy of 93.1%.

Jain et al. [29] trained deep-learning-based CNN models (i.e., ResNeXt, V3, and Xception) with chest X-ray scans. A dataset of chest X-rays (COVID-19 & Pneumonia) [30] was used that included categorized X-ray images of COVID-19. Compared to the other models, Xception reached the highest accuracy (i.e., 97.97%) for identifying chest X-ray scans.

In [31], Zouch et al. suggested a new method for automatically detecting COVID-19 in tomographic images (CT scans) and radiographic pictures (chest X-rays). Their research intended to develop an approach that could differentiate between COVID-19 and regular occurrences. Two databases were used, i.e., one containing CT scans [32], and the other one containing chest X-ray images [18]. The visual geometry group (VGG) and ResNet deep learning models improved the detection system’s precision for this pandemic. The results demonstrated that, for VGG19 and ResNet50, the proposed models reached an accuracy of 99.35 and 96.77% with all chest X-ray scans.

Kong et al. employed ResNet to separate effective image data to successfully categorize chest X-ray pictures in [33]. Two publicly available datasets were used [18,24]. Patients with pneumonia were divided into two categories for the study: patients with COVID-19 and those with common pneumonia. The results showed that the suggested model performed well in prediction. The ResNet model could recognize this binary categorization with a 98.0% average accuracy. The average accuracy for three-category categorization was 97.3%.

In [34], using machine vision approaches, Li et al. developed the Cov-Net CAD model for accurate COVID-19 classification. It focuses mostly on powerful and comprehensive feature-learning abilities. Two publicly available COVID-19 radiography datasets were used, i.e., [35,36]. The experimental results highlighted that the proposed Cov-Net was practical for the accurate recognition of COVID-19, with accuracy rates reaching 99.66% and 96.49% on issues involving three and four classes, respectively. Furthermore, the proposed Cov-Net surpassed six other well-known computer vision approaches under identical experimental conditions, demonstrating its excellence and competitiveness in developing highly discriminative features.

Table 1 displays comparison of recent related works on detecting the COVID-19 virus. The limitations of the recent related studies presented here are as follows:

- Both the training and testing of machine learning models were performed based on small databases with only a few X-ray images. Therefore, these methods would need more development before being applied.
- The number of multi-class datasets needs to be expanded so that models can effectively judge chest X-rays and give a more precise categorization diagnosis.
- Some deep learning models to identify COVID-19 suffer from overfitting and require a large network size. Furthermore, recent related efforts require many training parameters and complicated computer resources. As a result, they are difficult to deploy in practical applications, particularly in the healthcare field.

## 3. The Proposed Deep Learning Model

This section details the dataset used for training and testing, as well as the deep learning model proposed.

### 3.1. Dataset

**Figure 2:**
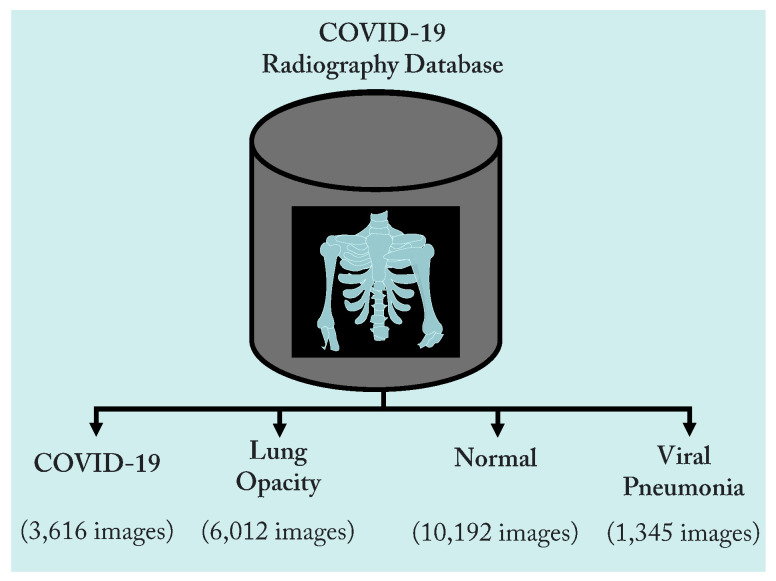
Classes and structure of the dataset.

**Figure 3:**
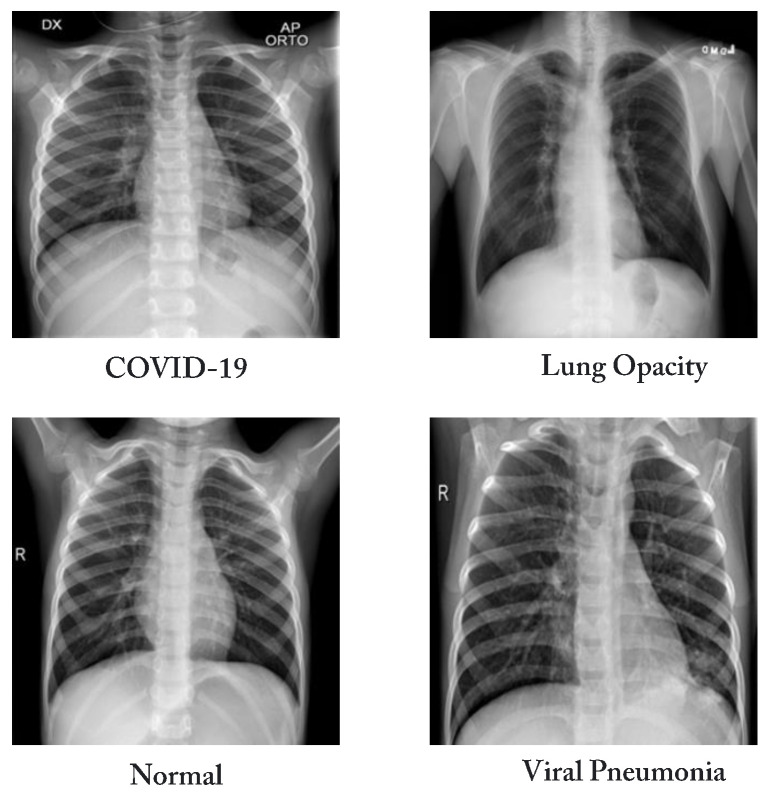
Examples of samples of dataset.

The COVID-19 Radiography dataset was used to train and assess the proposed technique. It was proposed by Rahman et al. and is freely available on Kaggle [37]. This dataset was revised three times, and for this study, we obtained the most recent version of the dataset. It contains 1345 images of viral pneumonia, 3616 chest X-ray images of COVID-19 infection, 10,192 chest X-ray images of normal cases, and 6012 scans of lung opacity, as shown in Figure 2. The dataset comprises several sub-datasets, falling into four distinct categories, i.e., COVID-19, lung opacity, normal, and viral pneumonia. Each class was selected from a different sub-dataset, and the dataset was generated by integrating several datasets. A total of 3616 images for the COVID-19 category were chosen from four different databases. With 2473 CXR images, the BIMCV-COVID19+ dataset [38] from the Valencian Region Medical Image Bank (BIMCV) significantly contributes to the existing set. It is one of the most comprehensive, publicly available independent databases. Other datasets that have COVID-19 data collections include the German Medical School dataset [39] (with 183 chest X-ray scans), SIRM, Kaggle, GitHub, and Twitter [40,41,42,43], which have 560 chest X-ray images. In addition, another dataset with 400 combined chest X-ray scans is accessible on GitHub [44]. Table 2 displays a description of the COVID-19 radiography DS in terms of classes, number of CXR scans, and sources. Figure 3 displays a sample from each category of the COVID-19 radiography classes.

### 3.2. The Structure of the COVID-ConvNet Model

**Figure 4:**
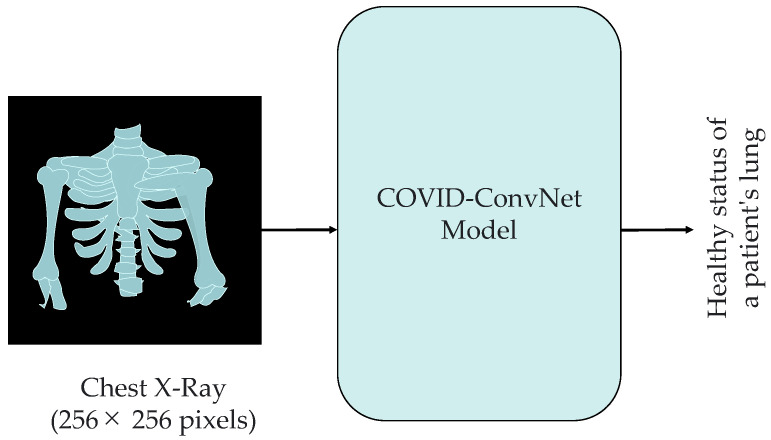
The proposed COVID-ConvNet model.

**Figure 5:**
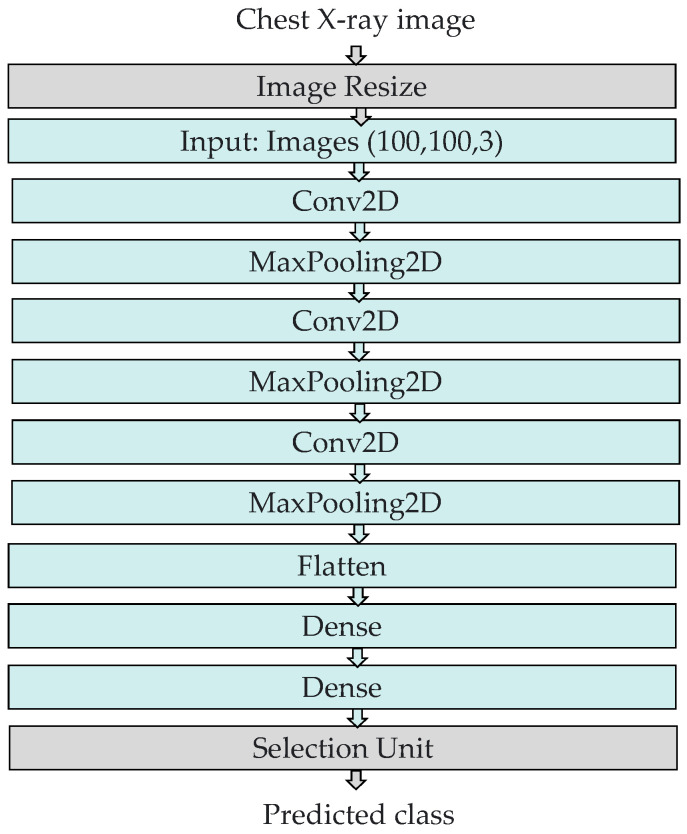
The structure of the proposed COVID-ConvNet model.

The proposed COVID-ConvNet has the ability to predict the health condition of a patient’s lung based on the processed dataset (Figure 4). In this article, we propose the performance of three experimental classifications with four classes (i.e., COVID-19, lung opacity, normal, and viral pneumonia), three classes (i.e., COVID-19, normal, and viral pneumonia), and two classes (i.e., COVID-19 and normal). As illustrated in Figure 5, convolution layers with maximum pooling layers, flattened layers, and thick layers make up the COVID-ConvNet model.

- Image resizing: The chest X-ray scans in the dataset had a size of 256 by 256 pixels. An image resizing process was performed to reduce the image size to 100 by 100 pixels.
- Convolution layers: All convolution layers were employed with a kernel size of (3, 3). In our study, the input shape of the CXR image was (100, 100, 3), where 100 denotes the width and height, while 3 indicates the input image’s three color channels (RGB). Rectified linear unit (ReLU), a piecewise linear function that returns a zero if the input is negative and returns the unchanged input value otherwise, served as the activation function of the convolution layers. ReLU is frequently employed as an activation function in convolution layers as it overcomes the vanishing gradient challenge, enabling the model to recognize characteristics more quickly and attain a high prediction performance. The filter size is 32 in the first convolution layer and gradually increases in the subsequent layers.
- Max pooling layers: These layers were employed to compress features to minimize calculation time [46]. We selected (2, 2) as the kernel size and stride in all of the convolutional network’s max pooling layers.
- Flatten layer: This layer generates a one-dimensional array vector from all pixels along the whole channels.
- Dense layers: The dense layer is a densely linked layer, entailing that every neuron of the dense layer acquires data from all neurons in the preceding layer. The activation function and units, which define the layer’s output size and element-wise activation in the dense layer, respectively, were the parameters employed by the dense layer. There were two dense layers at the end of our COVID-ConvNet model. The first one had a ReLU activation function, whereas the second one had a softmax activation function. The softmax activation function was utilized to forecast a multinomial probability distribution at the output layer.
- Selection unit: This unit was used to determine the index of the predicted class.

Hyperparameters are the settings or configurations of a machine learning model that are set prior to the training process. These settings can have a significant impact on the performance of the model, and the choice of hyperparameters can be critical for achieving good results. In our proposed COVID-ConvNet model, the following hyperparameters were utilized:

- Number of filters: The first convolutional layer employed a filter size of 32 to extract basic features from the input image. The subsequent convolutional layers had a filter size of 64 to capture more complex features and patterns from the output of the previous layer. This gradual increase in filter size allowed the network to learn increasingly complex representations of the input image, leading to better performance in classification tasks.
- Kernel size: The selected kernel size was (3, 3) for all the convolutional layers. This is a common choice for image classification tasks, as it allows the network to capture a range of features of different sizes. Additionally, using the same kernel size throughout the network ensures that the learned features are consistent across all layers, which can improve the network’s ability to generalize to new images.
- Stride: The stride in the given code was (2, 2) for all the max pooling layers. The stride determines the step size used when sliding the filter over the input image. A stride of (2, 2) means that the filter moves two pixels at a time in both the horizontal and vertical directions. Using a stride of (2, 2) can help to reduce the size of the output feature maps, which can help to reduce the computational cost of the network and prevent overfitting.
- Learning rate: The default learning rate was used, which was 1/1000 or 0.001. The learning rate is a hyperparameter that determines the step size used during the gradient descent to update the weights of the neural network. It is used because it is a reasonable starting point for many image classification tasks.
- Batch size: A batch size of 32 was used to determine the number of samples that are processed in each iteration of the training process. A batch size of 32 is a common choice for image classification tasks.

## 4. Experimental Analysis and Results

In this section, we present the experimental measures and results to demonstrate our COVID-ConvNet model’s ability to recognize COVID-19 instances from chest X-ray pictures.

### 4.1. Performance Metrics

A confusion matrix, sometimes referred to as a contingency table [47], was developed to evaluate the performance of a trained COVID-ConvNet model. A confusion matrix is indeed an effective tool for determining the ratios of true positives (TP), false positives (FP), true negatives (TN), and false negatives (FN) [48]. TP stands for “the number of samples projected and found to be positive”, while TN corresponds to “the number of samples predicted and found to be negative”. FP classification happens when a machine learning model classifies a sample as positive, but the target class appears to be negative. When a sample is initially classified as negative but later proven positive, the classification is FN [49]. Accuracy is defined as the ratio of the number of correctly categorized samples to the total number of testing samples [50,51], as described in Equation (1). The ratio of true positives to all positives is defined as precision (Equation (2)) [52,53]. Finally, the ratio of true positives to the total number of true positives and false negatives is defined as recall (Equation (3)). As indicated in Equation (4), the F-score is a combination of precision and recall [54,55].

(1) $$\begin{array}{c} \mathrm{Accuracy}=\frac{\mathrm{TP}\;+\;\mathrm{TN}}{\mathrm{TP}\;+\;\mathrm{FP}\;+\;\mathrm{TN}\;+\;\mathrm{FN}} \end{array}$$

(2) $$\begin{array}{c} \mathrm{Precision}=\frac{\mathrm{TP}}{\mathrm{TP}\;+\;\mathrm{FP}} \end{array}$$

(3) $$\begin{array}{c} \mathrm{Recall}=\frac{\mathrm{TP}}{\mathrm{TP}\;+\;\mathrm{FN}} \end{array}$$

(4) $$\begin{array}{c} F\text{-}\mathrm{score}=\frac{2\mathrm{TP}}{2\mathrm{TP}\;+\;\mathrm{FP}\;+\;\mathrm{FN}} \end{array}$$

### 4.2. Performance Results

Data samples were separated into two sub-sets: a training set to develop the proposed CNN model, and a testing set to assess models.The proportion of data allocated to the testing set was 20%, and the remaining 80% of the data were allocated to the training set. More specifically, the training and testing sets were composed of 16,932 and 4233 samples, respectively. To split the dataset into training and testing sets, a random shuffle method was used. This method is important to ensure that the data are not biased or ordered in a specific way that may affect the performance of the deep learning model. By randomly shuffling the data, we can reduce the risk of overfitting and ensure that the model is trained on a representative sample of the data. We present in this section the results for four, three, and two classes of classification for chest X-ray scans. To train and test the proposed COVID-ConvNet, the Google Colab platform was used, providing an NVidia Tesla K80 GPU with a single-core 2.3 GHz Xeon Processor, 320 GB of disk space, and 16 GB of RAM.

#### 4.2.1. Experiment 1: Four-Class Classification

**Figure 6:**
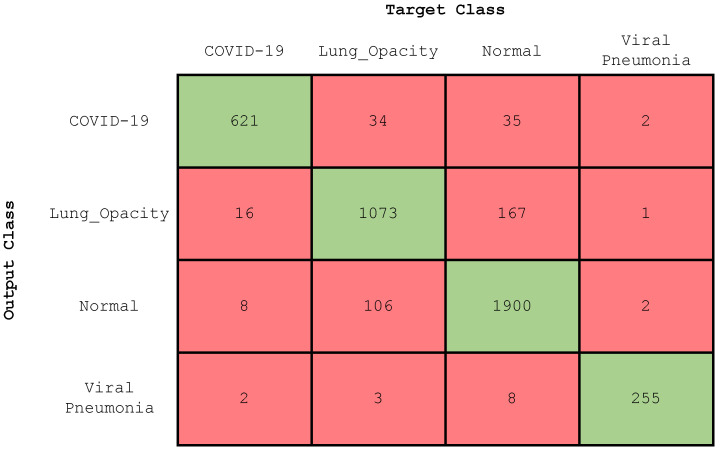
Confusion matrix (four-class classification).

In this section, we describe the results obtained for the four-class classification, i.e., COVID-19, lung opacity, normal, and viral pneumonia. Figure 6 illustrates the confusion matrix of the trained CNN model. After training for 50 epochs, our model reached an accuracy of 97.71%, 92.27%, 92.3%, and 99.57% on the testing subset for the COVID-19, lung opacity, normal, and viral pneumonia classes, respectively. Table 3 displays the evaluation values of the trained CNN with regard to accuracy, precision, recall, and F-score. As the table indicates, the evaluation metrics have been computed for each class separately according to Equations (1)–(4).

#### 4.2.2. Experiment 2: Three-Class Classification

**Figure 7:**
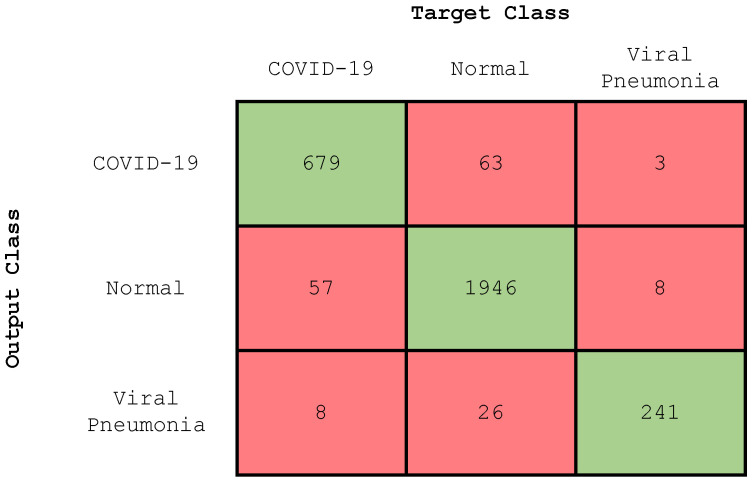
Confusion matrix (three-class classification).

In this section, we present the results of the three-class classification, i.e., COVID-19, normal, and viral pneumonia lungs. The confusion matrix of the trained CNN model is illustrated in Figure 7. Table 4 depicts the performance evaluation values for this experiment.

**Figure 8:**
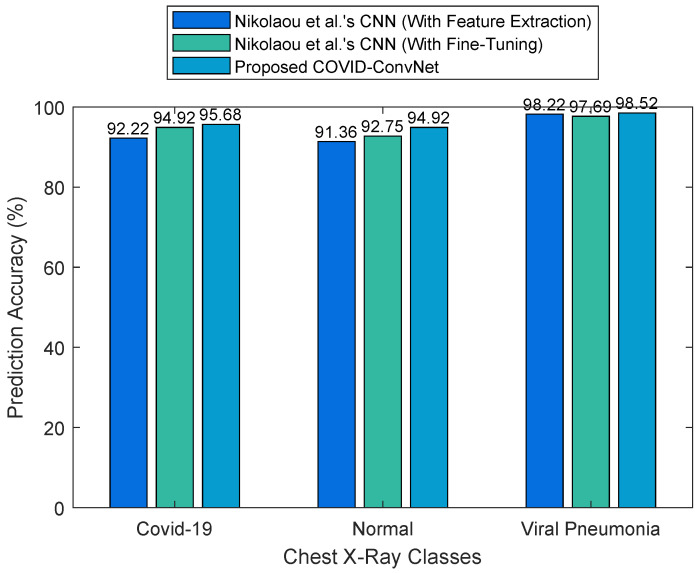
Comparison results of three-class classification.

Our COVID-ConvNet’s performance was further compared with Nikolaou et al.’s CNN models [21] in terms of the overall accuracy of the prediction. Figure 8 compares the COVID-19, normal, and viral pneumonia classification results. The results show that our proposed deep learning model achieved a higher accuracy than Nikolaou et al.’s CNN model with feature extraction by 3.66%, as well as higher results than their CNN model with fine-tuning by 1.88%.

#### 4.2.3. Experiment 3: Two-Class Classification

**Figure 9:**
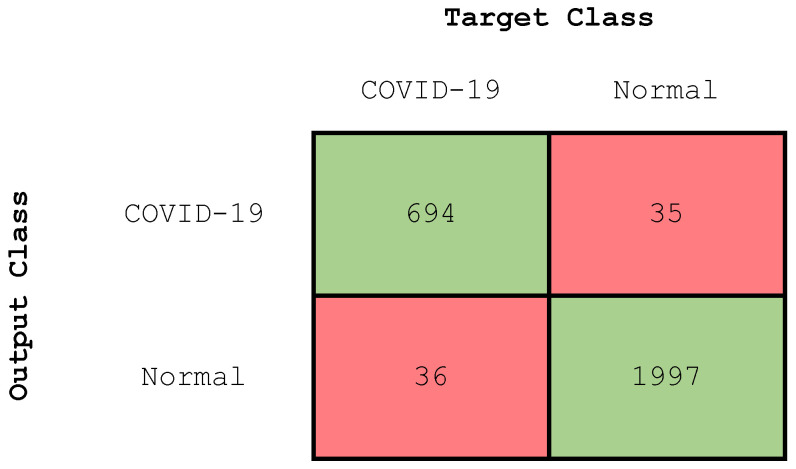
Confusion matrix (Two-class classification).

In this section, we present the results of the two-class classification problem (that is to say, COVID-19 vs. normal lungs). Figure 9 illustrates the confusion matrix of our CNN model, while Table 5 deals with the evaluation values.

**Figure 10:**
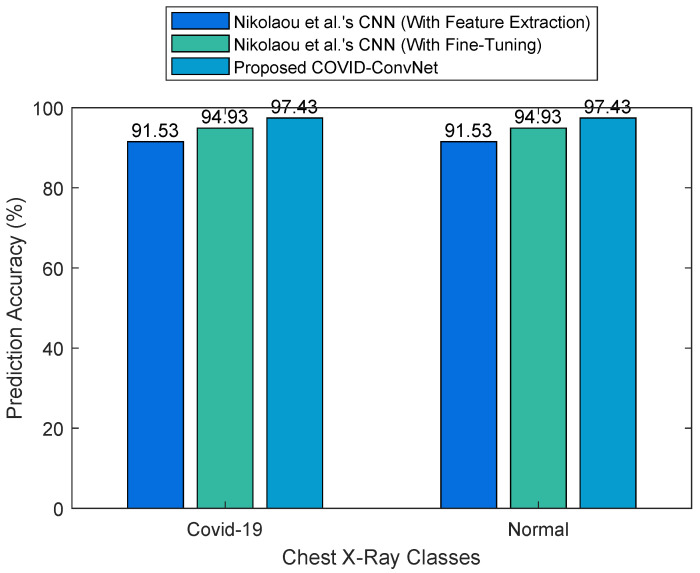
Comparison of two-class classification results.

Figure 10 represents a comparison of our results with Nikolaou et al.’s CNN models for the two classes of COVID-19 and normal. The results obtained show that our model outperformed Nikolaou et al.’s model with feature extraction by 5.9%, as well as their model with fine-tuning by 2.5%.

### 4.3. Considerations and Limitations of the COVID-ConvNet Model

While the use of the COVID-ConvNet model for COVID-19 screening and diagnosis has the potential to improve the accuracy and speed of diagnoses, there are several practical considerations and limitations that need to be considered. One practical consideration is the cost and availability of the necessary technology and expertise required to implement such a DL model in clinical settings. The use of the COVID-ConvNet model for COVID-19 screening would require access to high-performance computing resources and specialized expertise in deep learning and medical imaging. Another limitation of using CXR scans for COVID-19 detection is their lower sensitivity and specificity compared to other imaging modalities, such as CT scans. CXR scans are often used as a first-line screening tool for COVID-19 due to their lower cost and wider availability, but they may not always detect early or mild cases of the disease.

## 5. Conclusions and Future Work

As the COVID-19 pandemic continues to impact the world, the use of chest X-rays for diagnosis has become increasingly important. Convolutional neural networks have shown promising results in detecting COVID-19 from chest X-rays. In this article, we proposed an automatic detection model for COVID-19 infection based on chest X-ray scans called COVID-ConvNet. The Kaggle COVID-19 radiography dataset was selected to test and train the proposed COVID-ConvNet model because it offers a large and diverse collection of chest X-ray images (21,165 CXR scans) sourced from various repositories. Three experimental classifications were performed using the Google Colab platform, with four, three, and two classes. Experimental results showed that our deep learning model is superior to recent related works, reaching an accuracy of 97.43%. Furthermore, it outperforms Nikolaou et al.’s model with feature extraction and with fine-tuning in terms of prediction accuracy by up to 5.9%. It encourages multidisciplinary researchers to develop powerful artificial intelligence frameworks to combat the COVID-19 worldwide pandemic. The use of our proposed COVID-ConvNet model in COVID-19 patient screening has several potential implications. It can be applied in clinical practice for computer-aided diagnosis (CAD) systems to assist radiologists in interpreting medical images. These systems can help to improve the accuracy and speed of diagnoses, particularly in cases where the radiologist may be inexperienced, where the diagnosis is difficult, or in areas with limited access to PCR testing. Additionally, it can aid in the triage of patients, identifying those who require immediate medical attention, and it can assist in the monitoring of disease progression and response to treatment. In addition, combining CXR scans with other diagnostic tools such as laboratory tests, clinical examinations, and medical history can help to improve the accuracy of diagnoses and guide treatment decisions.

For future work, we aim to assess the performance of the COVID-ConvNet model using larger and more diversified datasets with more COVID-19 examples. In addition, feature engineering can be applied to enhance the performance of the deep learning model. Another direction for future research could be exploring the use of transfer learning to improve the performance of CNN models. Furthermore, we aim to investigate advanced techniques that can further address the issue of data imbalance and enhance the performance of our model. Specifically, we will explore methods such as data augmentation, class weighting, and resampling to balance the dataset and mitigate the impact of class imbalance. Another area for exploration could be the development of explainable AI techniques to provide insights into the decision-making process of CNN models. Finally, it would be valuable to investigate the generalizability of CNN models across different populations and imaging equipment. Overall, continued research in this area will be crucial in improving the accuracy and reliability of CNN-based COVID-19 detection from chest X-rays.

## Figures and Tables

**Table 1:** Comparison of recent related studies.

Ref.	Authors	Year	Number of Datasets Used	Type of Model Inputs	Number of Model Output Classes
[13]	Ohata et al.	2020	Two	CXR images	Two (COVID-19, normal)
[14]	Tabik et al.	2020	One	CXR images	Two (positive, negative)
[16]	Wang et al.	2020	One (compiled from five repositories)	CXR images	classes (normal, phenomena, COVID-19)
[17]	Hemdan et al.	2020	One	CXR images	Two (positive, negative)
[19]	Arias-Londoño et al.	2020	One	CXR images	Three (pneumonia, control, COVID-19)
[20]	Wang et al.	2020	One	CT images	Three (non-pneumonia, ILD, COVID-19)
[21]	Nikolaou et al.	2021	One	CXR images	Two (COVID-19, normal), Three (COVID-19, normal, viral pneumonia)
[22]	Ismael et al.	2021	One	CXR images	Two (COVID-19, normal)
[23]	Narin et al.	2021	Three	CXR images	Four (COVID-19, normal, viral pneumonia, bacterial pneumonia)
[26]	Abbas et al.	2021	Two	CXR images	Three (COVID-19, normal, SARS)
[29]	Jain et al.	2021	One	CXR images	Three (COVID-19, normal, pneumonia)
[31]	Zouch et al.	2022	Two	CT and CXR images	Two (COVID-19, normal)
[33]	Kong et al.	2022	Two	CXR images	Two (normal, pneumonia) Three (normal, pneumonia, COVID-19)
[34]	Li et al.	2022	Two	CXR images	Two (positive, negative), Three (COVID-19, normal, viral pneumonia), Four (COVID-19, normal, lung opacity, viral pneumonia)

**Table 2:** Description of COVID-19 Radiography dataset.

COVID-19 Radiography dataset [37]	Classes	Number of CXR scans	Sources
	COVID-19	3616	- BIMCV-COVID19+ dataset [38] (2473 CXR images).
			- German medical school [39] (183 CXR images).
			- SIRM, Github, Kaggle, Twitter [40,41,42,43] (560 CXR images).
			- Github source [44] (400 CXR images).
	Lung Opacity	6012	- Radiological Society of North America (RSNA) CXR dataset [45] (6012 CXR images).
	Normal	10,192	- RSNA [45] (8851 CXR images).
			- Kaggle CXR Images (pneumonia) database [24] (1341 CXR images).
	Viral Pneumonia	1345	- The CXR Images (pneumonia) database [24] (1345 CXR images).
Total number of CXR scans	21,165		

**Table 3:** Evaluation values of the trained CNN model (four-class classification).

Class	Accuracy (%)	Precision (%)	Recall (%)	F-Score (%)
COVID-19	97.71	90	96	93
Lung opacity	92.27	85	88	87
Normal	92.3	94	90	92
Viral pneumonia	99.57	95	98	97

**Table 4:** Evaluation values of the trained CNN model (three-class classification).

Class	Accuracy (%)	Precision (%)	Recall (%)	F-Score (%)
COVID-19	95.68	91	91	91
Normal	94.92	97	96	96
Viral pneumonia	98.52	88	96	91

**Table 5:** Evaluation values of the trained CNN model (two-class classification).

Class	Accuracy (%)	Precision (%)	Recall (%)	F-Score (%)
COVID-19	97.43	95	95	95
Normal	97.43	98	98	98

## Data Availability

In this work, we used a dataset called “COVID-19 Radiography Database”, which is available on Kaggle via the following URL: https://www.kaggle.com/tawsifurrahman/covid19-radiography-database (accessed on 1 Janurary 2023).

## Abbreviations

The following table gives a list of abbreviations used in our work:

AD-COVID19	Automatic detection of COVID-19
CAD	Computer-aided diagnosis
COVID-19	Coronavirus disease 2019
CNN	Convolutional neural network
COVID-ConvNet	COVID-19 convolutional network
COVID-SDNet	COVID-19-smart-data-based network
CT	Computed tomography
CXR	Chest X-ray
DeTraC	Decompose, transfer, and compose
DNN	Deep neural network
FN	False negative
FP	False positive
ILD	Interstitial lung disease
JSRT	The Japanese Society of Radiological Technology
MLP	Multi-layer perceptron
PCR	Polymerase chain reaction
PARL	Prior-attention residual learning
ReLU	Rectified linear unit
ResNet	Residual network
RGB	Red, green, and blue
RSNA	Radiological Society of North America
SARS	Severe acute respiratory syndrome
SVM	Support vector machine
TN	True negative
TP	True positive
VGG	Visual geometry group
WHO	The World Health Organization
